# dRiskKB: a large-scale disease-disease risk relationship knowledge base constructed from biomedical text

**DOI:** 10.1186/1471-2105-15-105

**Published:** 2014-04-12

**Authors:** Rong Xu, Li Li, QuanQiu Wang

**Affiliations:** 1 Medical Informatics Division, Case Western Reserve University, Cleveland, OH, USA; 2 Departments of Family Medicine and Community Health, Epidemiology and Biostatistics, Case Western Reserve University, Cleveland, OH, USA; 3 ThinTek LLC, Palo Alto, CA, USA

## Abstract

**Background:**

Discerning the genetic contributions to complex human diseases is a challenging mandate that demands new types of data and calls for new avenues for advancing the state-of-the-art in computational approaches to uncovering disease etiology. Systems approaches to studying observable phenotypic relationships among diseases are emerging as an active area of research for both novel disease gene discovery and drug repositioning. Currently, systematic study of disease relationships on a phenome-wide scale is limited due to the lack of large-scale machine understandable disease phenotype relationship knowledge bases. Our study innovates a semi-supervised iterative pattern learning approach that is used to build an precise, large-scale disease-disease risk relationship (D1 →D2) knowledge base (dRiskKB) from a vast corpus of free-text published biomedical literature.

**Results:**

21,354,075 MEDLINE records comprised the text corpus under study. First, we used one typical disease risk-specific syntactic pattern (i.e. “D1 due to D2”) as a seed to automatically discover other patterns specifying similar semantic relationships among diseases. We then extracted D1 →D2 risk pairs from MEDLINE using the learned patterns. We manually evaluated the precisions of the learned patterns and extracted pairs. Finally, we analyzed the correlations between disease-disease risk pairs and their associated genes and drugs. The newly created dRiskKB consists of a total of 34,448 unique D1 →D2 pairs, representing the risk-specific semantic relationships among 12,981 diseases with each disease linked to its associated genes and drugs. The identified patterns are highly precise (average precision of 0.99) in specifying the risk-specific relationships among diseases. The precisions of extracted pairs are 0.919 for those that are exactly matched and 0.988 for those that are partially matched. By comparing the iterative pattern approach starting from different seeds, we demonstrated that our algorithm is robust in terms of seed choice. We show that diseases and their risk diseases as well as diseases with similar risk profiles tend to share both genes and drugs.

**Conclusions:**

This unique dRiskKB, when combined with existing phenotypic, genetic, and genomic datasets, can have profound implications in our deeper understanding of disease etiology and in drug repositioning.

## Introduction

Phenomics, the systematic study of disease phenotypic relationships, is the natural complement to genomics in the post-genomic era [1-3]. Given the rapidly decreasing cost of genomics research, it has become clear that the bottleneck in understanding human disease has shifted dramatically from genetics to phenomics. Automatic approaches to obtaining and studying the observable disease-disease phenotypic relationships are critically important for unraveling both genetic and environmental mechanisms of complex diseases. Our long-term research goal is to develop encompassing and integrative systems approaches to both disease gene discovery and drug development by fully exploiting disease and drug data ranging from lower level genetic connections to immediate layer genomic data to higher level phenotypic data. While a large number of genetic and genomic datasets have been constructed to facilitate our understandings of the genetic mechanisms of diseases, large-scale disease phenotype datasets remain largely incomplete. Currently we are building a large-scale disease-phenotype relationship knowledge base from multiple heterogeneous and complementary sources including published biomedical literature, patient electronic health records (EHRs), and biomedical ontologies. This disease-phenotype relationship knowledge base will include relationships such as disease-risk (i.e. disease-associated genes, environmental risk factors, and other predisposing diseases), disease co-morbidity, disease-organ, and disease-manifestation relationships, among others. As part of our ongoing effort, this study focuses on building a large-scale disease-disease risk relationship knowledge base (dRiskKB) by extracting risk-specific disease pairs (i.e. obesity →type 2 diabetes, hypertension →stroke) from over 21 million MEDLINE records.

We recently developed a knowledge-driven pattern-learning approach to automatically extract disease-manifestation (symptom) pairs from biomedical literature [4]. In that study, we leveraged the large amount of external knowledge from biomedical ontologies (50,543 disease-manifestation pairs defined in the Unified Medical Language System (UMLS) semantic network in order to discover disease-manifestation-specific syntactic patterns. Using the learned patterns, we extracted a total of 121,359 disease-manifestation pairs from MEDLINE, the majority of which had not been captured in ontologies. Unlike our previous work, which leveraged a large number of known disease-manifestation pairs from biomedical ontologies as prior knowledge, this current study did not benefit from prior knowledge, because no knowledge base of disease-disease risk pairs currently exists. To circumvent the problem, we developed a semi-supervised iterative pattern learning approach to automatically discover disease-risk-specific syntactic patterns. This semi-supervised approach requires no external knowledge and takes a single pattern as the seed. To the best of our knowledge, this is the first large-scale effort to build a disease-disease risk relationship knowledge base from the vast amount of published biomedical literature. The main contributions of our study are two-fold: First, we develop an efficient and effective semi-supervised approach to automatically find textual patterns that specify risk-specific semantic relationships between diseases. Second, we build the dRiskKB, a large-scale knowledge base of disease-disease risk relationships. This unique disease-phenotype relationship knowledge base, when combined with existing phenotypic, genetic, and proteomic datasets, can have profound implications in our deeper understanding of disease etiology and in rapid drug repositioning.

## Background

Perplexing relationships among diseases often go unexplained. How, for example, does obesity contribute to cancer risk? Why are patients with certain neurological diseases, like Parkinson’s, at lower risk for many cancers? Understanding the genetic and environmental factors responsible for these striking risk-specific relationships among diseases may reveal novel insights into the molecular mechanisms of disease development and lead to better disease prevention and treatment. It has been increasingly recognized that phenotypically-related diseases often reflect overlapping molecular causation [5-9]. Recently, disease phenotypic similarity has become another major data source exploited by computational methods in discovering novel candidate disease genes [10-17]. The advantage of this phenotype-driven approach over traditional approaches is that we can hypothesize that similar phenotypes in two diseases may result from genes/pathways that are involved in the same biological processes. For phenotype-driven candidate gene selection, a two-layered heterogeneous data network is often constructed where the phenotypic network layer consists of connections between similar diseases, while the genetic network layer contains molecular data such as protein-protein interaction (PPI), pathways, gene co-expression, or shared protein domain. These two network layers are then linked through known disease-gene associations [13]. Currently, disease phenotype networks are mainly constructed based on disease co-morbidity [18] or text mining of the Online Mendelian Inheritance in Man (OMIM) database [10-19]. For systems approaches to studying phenotypic relationships among diseases, disease-disease phenotypic associations (i.e. disease-manifestation, disease-risk, disease-organ, disease-comorbidity) from multiple heterogeneous sources (i.e. published literature, patient EHRs, and biomedical ontologies) are necessary to mitigate the incompleteness and biases inherent to many biomedical networks [20]. In this study, we focus on building a disease-risk relationship knowledge base by automatically extracting disease-disease risk pairs from MEDLINE.

Currently, more than 21 million biomedical records are available on MEDLINE, making it an excellent source of disease-risk knowledge. For example, searching PubMed for the phrase “is a risk factor for” returns a total of 52,460 sentences, among which more than 6,000 sentences are related to cardiovascular disease risks, and another 6,000 are related to diabetes risks. By the same token, the single sentence “**Obesity ***is a risk factor for ***colorectal cancer**, and **hyperinsulinemia**, a common condition in obese patients, may underlie this relationship” (PMID 18172327) contains the observed risk relationship among three diseases: *obesity*, *colorectal cancer* and *hyperinsulinemia*. Despite the rich disease risk-specific semantic relationship knowledge contained in this corpus of published biomedical literature, the fact that the knowledge is buried in free text with limited machine understandability poses a significant barrier.

Automatic extraction of biomedical relationships from MEDLINE is a highly active area of research. Common approaches for biomedical relation extraction use rule-based, co-occurrence-based statistical approaches or natural language processing (NLP) approaches. These have most often been applied to extract relationships between drugs, diseases, proteins, and genes [21-24]. Research efforts in disease-risk relationship extraction tasks, however, have been quite limited. Recently, Liu et al. manually identified environmental etiological factors associated with 3,159 diseases by searching the MeSH annotations of MEDLINE articles [25]. Fiszman et al. extracted risk factors for Metabolic Syndrome from medical literature by using the MeSH heading “Risk Factors” to retrieve risk-specific sentences [26]. While Liu’s study is based on manual curation, Fiszman’s study falls more under the supervised machine learning approach category since it relies on the semantic relationships available in the Unified Medical Language System (UMLS) semantic network and only focuses on one type of disease: Metabolic Syndrome. In addition, studies have shown that using manually assigned MeSH terms, as in both the above-mentioned studies, results in limited recall in categorizing biomedical articles [27]. Currently, no risk-specific disease-disease semantic relationship knowledge base exists that can be leveraged upon in developing computational approaches to both disease gene discovery and drug development.

## Approach

Automatically extracting disease-risk relationships from free text is a challenging task. Risk factors for diseases are often complicated and highly heterogeneous, including genes (e.g. APOE, BRCA1), predisposing diseases (e.g. hypertension, hypogonadism, obesity), chemicals (e.g. exposure to benzene, estrogen, aflatoxins), life styles and behavior (e.g. smoking, alcohol use, physical inactivity, excess salt intake), family history, ethnicity, age, and gender, among many other factors. No specific lexicon of disease-associated risk factors exists, yet such an entity is required by most information extraction systems for relationship extraction. Even our current task of extracting risk-specific relationships among diseases is difficult. In general, extracting specific semantic relationships among the same type of entities, such as disease-co-morbidity, disease-manifestation, and disease-disease risk pairs, is more challenging than extracting relationships between two different types of entities, such as drug-disease, drug-gene and drug-side effects.

Recent studies in semi-supervised pattern learning approaches are motivated by the use of a very large collection of texts (web) [28]. Since semi-supervised approaches have the advantage of requiring minimal human annotation of data, they are able to extract broad types of relationships from free text. Semi-supervised learning approaches have been used in non-biomedical domains to extract information from the web [29-35]. However, the potential use of semi-supervised approaches to build large-scale biomedical databases that enable systems approaches to disease gene discovery and drug repositioning has not been fully explored.

Recently, we developed a series of semi-supervised pattern learning approaches for named entity recognition [36,37], relationship extraction [38], and medical image retrieval from the web [39]. Semi-supervised learning approaches depend on the regularity of language and the redundancy of data. A big corpus such as MEDLINE is ideal for such tasks. In our current study, we develop an efficient and effective semi-supervised pattern-learning algorithm to extract disease-disease risk relationships from MEDLINE. Since our ultimate research goal is to develop systems approaches that exploit disease-phenotype relationships for subsequent network-based candidate gene prediction and drug repositioning, the precision and scalability of the relationship extraction algorithms is critical. Pattern-based relationship extraction approaches have the advantage of being highly precise and efficient. In addition, since our approach is semi-supervised, it has the advantage of requiring minimal human intervention and no external domain knowledge.

## Methods

### Build a local MEDLINE search engine

We downloaded a total of 21,354,075 MEDLINE citations (119,085,682 sentences) published between 1965 and 2012 from the U.S. National Library of Medicine (http://mbr.nlm.nih.gov/Download/index.shtml). Each sentence was syntactically parsed with Stanford Parser [40] using the Amazon Cloud computing service (a total of 3,500 instance-hours with High-CPU Extra Large Instance used). We used the publicly available information retrieval library Lucene (http://lucene.apache.org) to create a local MEDLINE search engine with indices created on both sentences and their corresponding parse trees.

### Build a clean disease lexicon

A highly accurate and comprehensive disease lexicon is critical for the task of building a high quality disease-phenotype relationship knowledge base, including our current task of building dRiskKB. We recently built a large clean disease lexicon by combining and manually cleaning all disease terms from UMLS with the following semantic types: “Disease Disease or Syndrome”, “Sign or Symptom”, “Neoplastic Process”, “Congenital Abnormality”, “Mental or Behavioral Dysfunction” and “Anatomical Abnormality”, and from the Human Disease Ontology (http://bioportal.bioontology.org/ontologies/1009). We used this clean disease lexicon in our recent study of extracting disease-manifestation [4] and drug-disease pairs [24] from MEDLINE. The clean disease lexicon consists of a total of 70,247 disease terms (corresponding to 28,540 distinct disease concepts) that appear in MEDLINE. The cleaned disease lexicon was manually curated by curators from ThinTek.com and can be obtained for free academic use by contacting co-author qwang@thintek.com.

### Semi-supervised disease-disease risk relationship extraction

**Figure 1:**
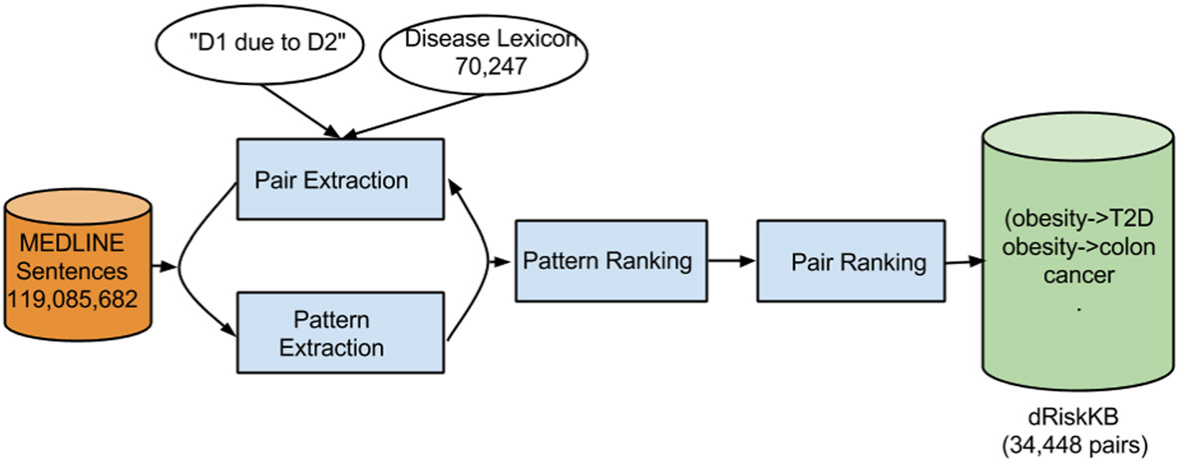
The semi-supervised pattern-learning approach for extracting disease-disease risk pairs from MEDLINE.

The semi-supervised relationship extraction algorithm is depicted in Figure 1 and can be formulated as follows: Given: (1) a seed pattern such as “*D1 due to D2*” where both *D1* and *D2* are disease terms from the input disease lexicon; (2) a text corpus of MEDLINE sentences and their corresponding parse trees; (3) a disease lexicon. Do: starting from the seed pattern, which represents a typical expression of a disease-disease risk relationship, iteratively discover new patterns and extract new pairs with newly discovered patterns. When no significant number of new patterns is discovered, rank extracted patterns and pairs.

#### *Pair Extraction*

Seed pattern or patterns extracted from previous iterations were used as search queries to the local MEDLINE search engine. Both sentences and parse trees that contained these patterns were retrieved. We extracted disease-disease pairs from the retrieved sentences if the disease pairs and the pattern followed the restriction: “*D1 pattern D2*,” wherein both D1 and D2 are disease terms as well as noun phrases in the retrieved sentences. The requirement that both diseases be noun phrases in the sentences was formulated to avoid false positives. For example, without this restriction, the incorrect (or incomplete) D1 →D2 pair “*infection →hypertension*” would be extracted from the following sentence: “*Herpes simplex virus type 2 ***infection ***is a risk factor for ***hypertension**” (PMID 15492472), since the disease term “*infection*” instead of the more specific term “*Herpes simplex virus type 2 infection*” is included in the input disease lexicon. The correct D1 →D2 risk pair in above sentence is “*Herpes simplex virus type 2 infection →hypertension*”, not “*infection →hypertension*.”

#### *Pattern extraction*

Disease pairs (D1-D2) extracted from the previous iteration were used as search queries to the local MEDLINE search engine. Corresponding sentences and parse trees were retrieved. Syntactic patterns between the disease pairs were extracted if D1-D2 pairs and their patterns conformed to the following format: “*D1 pattern D2*,” wherein both diseases *D1* and *D2* were noun phrases in the retrieved sentences.

#### *Pattern ranking*

The iterative pair extraction and pattern extraction processes ran until no significant number of new patterns was discovered (two iterations in this study). We then ranked extracted patterns. Each pattern was ranked based on how similar its output (its associated D1-D2 pairs) was to the output of the seed pattern. Using the output of the seed pattern (*p*0) as the gold standard, we developed three pattern-ranking algorithms: (1) *Precision-based* ranking, wherein patterns were ranked based on pattern specificity; (2) *Recall-based* ranking, wherein patterns were ranked based on pattern generality; and (3) *F1-based* pattern ranking, wherein both pattern specificity and generality were taken into account. We define *ins*(*p*) to be the set of pairs matched by pattern p, and the intersection *ins*(*p*) ∩ *ins*(*p*0) as the set of pairs matched by both pattern *p* and seed pattern *p*0. The *Precision-based*, *Recall-based*, and *F1-based* ranking scores are defined as below:

(1) $$\begin{array}{ll} {\text{RS}}_{\text{precision}}(p) & =\frac{\left|\text{ins}(p)\cap \text{ins}(p0)\right|}{\left|\text{ins}(p)\right|}\; \end{array}$$

(2) $$\begin{array}{ll} {\text{RS}}_{\text{recall}}(p) & =\frac{\left|\text{ins}(p)\cap \text{ins}(p0)\right|}{\left|\text{ins}(p0)\right|}\; \end{array}$$

(3) $$\begin{array}{ll} {\text{RS}}_{F1}(p) & =\frac{2*\text{precision}(p)*\text{recall}(p)}{\text{precision}(p)+\text{recall}(p)}\; \end{array}$$

#### *Pair ranking*

Extracted pairs were ranked based on both the scores of their associated patterns and their frequency counts in MEDLINE. A reliable D1 →D2 pair is one that is associated with reliable patterns many times. The ranking score (RS) of a pair or a relationship (R) is defined as follows:

(4) $$\text{RS}(R)=\overset{n}{\underset{i=0}{\sum }}\text{log}(\text{RS}(P_{i}))*\text{count}(P_{i},R)$$

*RS*(*P*_*i*_) is the score of its associated patterns (*P*_*i*_), which is defined in (1), (2), or (3), and *count* (*P*_*i*_,*R*) is the number of times that the pair is associated with the pattern in the entire MEDLINE corpus.

### Pattern selection, database construction and manual evaluation

From the top-ranked patterns (based on F1-based pattern ranking method), we manually selected a total of 26 disease risk-specific patterns that associated with at least 100 unique disease-disease risk pairs. These patterns had both high precisions and recalls as determined by how they ranked and by the manual examination. The manual examination of top-ranked patterns took about 15 minutes. We then extracted disease-disease risk pairs from MEDLINE associated with these patterns. These pairs were used in in building dRiskKB database.

Using each of the 26 selected patterns and their associated disease-disease risk pairs as search queries to the local MEDLINE search engine, we retrieved sentences that contained these patterns and disease pairs in the format of “D1 pattern D2.” From these retrieved sentences, we randomly selected 50 sentences for each pattern (a total of 26*50 = 1300 sentences) for manual curation. Two annotators independently curated these sentences. Disease-disease risk pairs from these sentences were classified to one of three categories: correct, partially correct, and incorrect. Precisions of patterns as well as of pairs were calculated using pairs that were identified as correct by both annotators; these functioned as the gold standard. The kappa statistics that measures the agreement between the two annotators [41] was as high as 0.95.

### Systematically analyze extracted disease-disease risk (D1 →D2) pairs

#### *Analyze the correlation between disease-risk relationships and disease-associated genes*

We analyzed shared the genetic components underlying the direct D1 →D2 risk pairs. We also analyzed the shared genes for disease-disease (D1-D2) pairs with overlapping risk diseases (D1 ←{d11, d12, …, d1n}, D2 ← {D21, d22, …, d2m}) or D1-D2 pairs with overlapping effect diseases (D1 →{d11, d12, …, d1n}, D2 →{d21, d22, …, d2m}). We used two complementary sources of disease-gene association knowledge for this analysis. The first one was from the OMIM (Online Mendelian Inheritance in Man) (data accessed in 04/2012) [42], and consisted of 14,870 pairs for 2,391 diseases and 8,929 genes. The second was from the NHGRI’s (National Human Genome Research Institute) GWAS Catalog database (data accessed in 01/2012) [43] and consisted of 5,895 disease/trait-gene pairs for 520 diseases and 3,795 genes. OMIM is a database that catalogues many Mendelian diseases with known genetic components. The GWAS Catalog database is an online database of SNP-trait associations derived from genome-wide association studies. Many diseases from the GWAS catalog are common complex diseases such as hypertension and diabetes. We first mapped disease terms between the extracted D1 →D2 pairs and the disease-gene pairs from OMIM and from the GWAS catalog. We calculated the average number of shared genes between disease-disease pairs (D1 →D2 risk pairs, D1-D2 pairs with shared predisposing disease, or D1-D2 pairs with shared effect diseases) and compared the number to all disease-disease pairs for mapped diseases. For disease-disease pairs that shared risk diseases or effect diseases at different cutoffs, we calculated the average number of shared genes at each cutoff.

#### *Analyze the correlation between disease-risk relationships and disease-associated drugs*

Similarly, we analyzed the shared drug treatments for direct D1 →D2 risk pairs, for D1-D2 pairs with shared predisposing diseases, and for D1-D2 pairs with shared effect diseases. We extracted a total of 52,000 disease-drug treatment pairs from ClinicalTrials.gov (http://www.clinicaltrials.gov/), a registry of federally and privately supported clinical trials conducted in the United States and around the world (http://www.clinicaltrials.gov), as the disease-drug association knowledge. These disease-drug pairs consisted of 9,591 diseases and 2,035 drugs. We mapped the disease terms between. These disease-drug pairs consisted of 9,591 diseases and 2,035 drugs. We mapped the disease terms between disease-drug pairs and disease-disease risk pairs. As in the above genetic correlation study, we calculated the average number of shared drugs between D1 →D1 risk pairs and compared it to that of all disease-disease pairs. For disease-disease pairs that shared risk diseases or effect diseases at different cut-offs, we calculated the average number of shared drugs at each cutoff.

## Results

### Top ranked patterns contain many disease risk-specific patterns

Using the typical disease risk-specific pattern “D1 due to D2” as a search query to the local MEDLINE search engine, we retrieved a total of 22,482 sentences containing two diseases and the seed pattern in the format of “D1 due to D2,” wherein both D1 and D2 were diseases and also noun phrases in the sentences. From these sentences, we extracted a total of 14,183 unique D1 ←D2 pairs (“Pair Extraction”). We then used these extracted D1 ←D2 pairs as search queries to the local MEDLINE search engine to find their associated patterns (“Pattern Extraction”). After two iterations, we stopped the process since not many additional risk-specific patterns with both high precision and high recall were discovered. We then ranked the extracted patterns (a total of 2,119,091) using the output associated with the seed pattern as the gold standard (the 14,183 D1 ←D2 pairs associated with the seed “*D1 due to D2*”). The three pattern-ranking methods are *Precision-based*, *Recall-based* and *F1-based*. Top 10 ranked patterns for each method are listed in Table 1 (patterns for D1 ←D2 relationship) and in Table 2 (patterns for D1 →D2). The risk-specific patterns associated with at least 1000 distinct disease-disease risk pairs are highlighted.

**Table 1:** Top 10 ranked patterns and numbers of associated D1 ←D2 pairs

**Precision-based**		**Recall-based**		**F1-based**	
**Pattern**	**Pairs**	**Pattern**	**Pairs**	**Pattern**	**Pairs**
“**D1 due to D2**”	14,183	“**D1 due to D2**”	14,183	“**D1 due to D2**”	14,183
“D1 was due to D2”	198	“D1 and D2”	205,942	“**D1 caused by D2**”	8,297
“D1 owing to D2”	175	“D1 in D2”	50,902	“**D1 secondary to D2**”	6,499
“D1 attributable to D2”	279	“**D1 caused by D2**”	8,297	“D1 from D2”	7,993
“D1 was caused by D2”	181	“D1 associated with D2”	27,477	“D1 associated with D2”	27,477
“D1 due to chronic D2”	146	“**D1 secondary to D2**”	6,499	“D1 in patients with D2”	20,221
“D1 due to severe D2”	187	“D1 in patients with D2”	20,221	“D1 in D2”	50,902
“D1 as a result of D2”	516	“D1 with D2”	35,203	“D1 of D2”	11,919
“**D1 resulting from D2**”	1,281	“D1 from D2”	7,993	“**D1 resulting from D2**”	1,281
“D1 attributed to D2”	184	“D1, D2”	99,881	“D1 related to D2”	1,616

Risk-specific patterns associated with **≥**1000 pairs are highlighted.

**Table 2:** Top 10 ranked patterns and numbers of associated D1 →D2 pairs

**Precision-based**		**Recall-based**		**F1-based**	
**Pattern**	**Pairs**	**Pattern**	**Pairs**	**Pattern**	**Pairs**
“**D1 due to D2**”	14,183	“**D1 due to D2**”		14,183 “**D1 due to D2**”	14,183
“D1 is a leading cause of D2”	132	“D1 and D2”	205,942	“D1 with D2”	35,203
“D1 is the most common cause of D2”	188	“D1 with D2”	35,203	“D1 D2”	12,887
“D1 is a major cause of D2”	281	“D1, D2”	99,881	“**D1 causing D2**”	2,260
“D1 is the main cause of D2”	104	“D1 D2”	12,887	“D1 patients with D2”	2,578
“D1 is a frequent cause of D2”	104	“D1 or D2”	38,841	“**D1 as a cause of D2**”	1,463
“D1 is an important cause of D2”	262	“D1 in D2”	50,902	“D1 without D2”	3,703
“D1 is a common cause of D2”	351	“D1 associated with D2”	27,477	“**D1 complicated by D2**”	3,422
“D1 as cause of D2”	117	“D1, and D2”	28,942	“D1 or D2”	38,841
“D1-induced D2”	558	“**D1 causing D2**”	2,260	“D1 and D2”	205,942

Risk-specific patterns associated with ≥1000 pairs are highlighted.

As shown Table 1, the *Precision-based* method was able to rank disease-risk-specific patterns highly on the list. For example, all of the top 10 patterns as determined by the method *Precision-based* are risk-specific patterns, including “*D1 owing to D2*,” “*D1 attributable to D2*,” and “*D1 was caused by D2*.” However, the majority of these top-ranked patterns were associated with less than 1000 disease pairs; the exception is the pattern “*D1 resulting from D2*,” which was associated with 1,281 pairs. On the other hand, top patterns ranked by the *F1-based* method included more risk-specific patterns with high recalls. For example, the second highest ranking pattern, “*D1 caused by D2*,” was associated with a total of 8,297 distinct D1 ←D2 pairs. The third highest ranking pattern, “*D1 secondary to D2*,” was associated with 6,499 distinct D1 ←D2 pairs. The *Recall-based* method performed similarly to the *F1-based* ranking method in ranking many risk-specific patterns with high recalls highly.

Using the same 14,183 D1 ←D2 pairs associated with the seed pattern “D1 due to D2” as the gold standard, we also learned typical patterns that specify risk relationship in the reverse direction (D1 →D2), such as “*D1 causing D2*,” and “*D1 as a cause of D2*.” As shown in Table 2, all top 10 patterns ranked by the *Precision-based* method are disease risk-specific patterns such as “*D1 is a leading cause of D2*” and “*D1 as a cause of D2*.” Even though these top-ranked patterns are highly specific, they had limited recalls in that each of them was associated with less than 1000 D1 →D2 pairs. Both the *F1-based* and *Recall-based* methods were able to rank risk-specific patterns with high recalls at the top. For example, two additional risk-specific patterns with high recalls appeared in the top 10 patterns ranked by the *F1-based* approach: “*D1 causing D2*,” (2,260 pairs) and “*D1 as a cause of D2*” (1,463 pairs).

### Disease-disease risk pairs extracted using the selected patterns are of high precision

**Figure 2:**
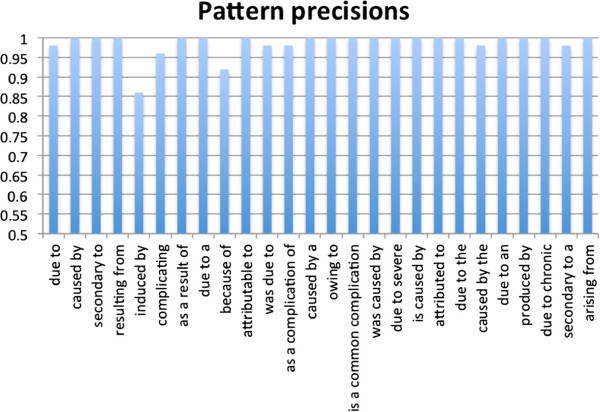
Pattern precisions.

We manually examined a total of 1,300 (50 for each of the 26 selected patterns) randomly selected sentences that contained patterns and their associated disease-disease risk pairs in the format “D1 pattern D2.” A pattern is correct for a given sentence if the semantic relationship between its associated disease-disease pairs is disease-risk-specific. As shown in Figure 2, all the selected patterns were highly precise, with an average precision of 0.99.

We then calculated the precisions of disease pairs associated with these patterns. The correctness of the pairs depends on not only their associated patterns but also the underlying disease lexicon and the parsing accuracy of Stanford parser. From the 1,300 sentences, we extracted a total of 1,203 distinct mention-level disease-disease pairs, among which 1,085 pairs were correct with a precision of 0.919 and 1,185 pairs were partially correct with a precision of 0.985. The high precision of the extracted pairs reflects that the specificity of the selected patterns, the accuracy of the manually curated input disease lexicon, and the strategy of using parse trees to enforce the rule that disease terms be noun phrases in the sentences. The majority of partially incorrect extracted disease pairs was caused by the way we delineated the noun phrase boundary. For example, from the sentence “**Sebaceous carcinoma ***arising from ***Bowen’s disease** of the vulva” (PMID 3767405), the partial pair “Sebaceous carcinoma-Bowen’s disease” was extracted, rather than the more complete pair ‘Sebaceous carcinoma-Bowen’s disease of the vulva”. The disease term “bowen’s disease” not “bowen’s disease of the vulva” is included in the disease lexicon. In addition, “bowen’s disease” by itself is a noun phrase in the parse tree of the sentence: (ROOT (NP (NP (JJ sebaceous) (NN carcinoma)) (VP (VBG arising) (PP (IN from) (NP **(NP (NP (NN bowen) (POS ’s)) (NN disease))** (PP (IN of) (NP (DT the) (NN vulva)))))).

In our current study, we learned many patterns after two iterations; however, many of these patterns are similar such as “due to” and “was due to.” One of the limitations of our study is that we did not merge similar pattern since we did not have an automatic way to do this systematically. However, this limitation should not have affected our evaluation results. The majority of the 26 selected patterns are distinctive, such as “due to”, “caused by”, and “secondary to”. In addition, the results across these 26 patterns are very consistent.

### Pattern extraction and ranking algorithms are robust in terms of seed patterns

Since the performance of many bootstrapping iterative pattern-learning systems may depend on the choice of initial seeds, an important question is whether these different starting points lead to different results. We investigated this issue by starting from five different seed patterns and examined whether the 26 selected risk-specific patterns that were selected from top-ranked patterns of the seed pattern “D1 due to D2” are also enriched in top ranked patterns when other seeds are used.

The five seed patterns include three typical risk-specific patterns with both high precisions and recalls:“D1 due to D2” (seed1), “D1 caused by D2” (seed2), and “D1 secondary to D2” (seed3), a risk-specific pattern with high precision but relatively low recall: “D1 attributable to D2” (seed4), and a non-risk-specific general pattern: “D1 and D2” (seed5). We ran the iterative pattern extraction and relationship extraction for two iterations using each of the five patterns as the seed. After two iterations, we ranked the extracted pattern using the F1-based pattern ranking method and counted how many of the 26 risk-specific patterns appeared among top-ranked pairs at 10 different ranking cutoffs (top 10, 20, …, 100). As shown in Table 3, the outputs (as measured by the appearance of the 26 risk-specific patterns) among three seed patterns with both high precisions and recalls (seed1, seed2 and seed3) are similar. For example, 24 of the 26 risk-specific patterns appeared in the top 100 patterns for seed “D1 caused by D2”. Top-ranked patterns for the relatively more specific risk-specific pattern (“D1 attributable to D2”) are also enriched with patterns from the 26 selected patterns, however, the enrichment is smaller compared to those for seed1, seed2 and seed3. As a negative control, we also used a non-risk-specific pattern “D1 and D2” as seed. The enrichment of disease-specific patterns among top ranked patterns for this non-risk-specific seed is much smaller compared to those of other four seeds. In summary, the pattern-ranking algorithm is robust in terms of seed choices as long as the seed is a risk-specific pattern with relatively high precision and recall (aka, a typical pattern specifying the risk relationship among diseases) is used. We experimented in using more than one patterns as seeds, and found that the algorithm is not sensitive to the number of seed patterns used (data not shown).

**Table 3:** Number of disease-risk-specific patterns among top-ranked patterns for five different seeds: seed1 (“D1 due to D2”), seed2 (“D1 caused by D2”), seed3 (“ D1 secondary to D2”), seed4 (“ D1 attributable to D2”), and seed5 (“D1 and D2)”

**Rank**	**Seed1**	**Seed2**	**Seed3**	**Seed4**	**Seed5**
10	4	4	4	5	1
20	6	7	7	11	3
30	8	11	11	11	3
40	12	15	15	12	4
50	16	20	15	15	4
60	21	21	17	15	4
70	23	22	21	16	4
80	25	23	22	16	5
90	26	24	23	16	5
100	26	24	23	16	5

### Disease-disease risk pairs tend to share both genes and drugs

The F1-based ranking method prioritized many risk-specific patterns with both high precisions and high recalls on the top; however not all top-ranked patterns were necessarily disease-risk-specific patterns. We manually selected 26 risk-specific patterns with high recalls from the top-ranked patterns and then extracted disease-disease risk pairs from MEDLINE using these selected patterns. Examples of these selected patterns are “*D1 due to D2*,” “*D1 caused by D2*,” “*D1 secondary to D2*,” and “*D1 resulting from D2*.” Using these patterns, we extracted a total of 34,448 unique disease-disease risk pairs (D1 ←D2), representing 12,981 diseases. We then analyzed the relationships between these 34,448 disease-disease risk pairs and disease-related genes and drugs (Table 4).

**Table 4:** Percentages of disease-disease risk pairs (D1 →D2) that share any genes or drugs (Column 2), the average numbers of shared genes or drugs for D1 →D2 pairs (Column 3), and the average numbers of shared genes or drugs for disease-disease combinations (D1-D2) (Column 4)

**Source**	**Percent**	**Average**	**Average**
	**(D1 *→ *** **D2)**	**(D1 *→*** **D2)**	**(D1-D2**
			**combinations)**
Disease-gene (OMIM)	3.79%	**5.36**	0.016
Disease-gene (GWAS)	13.64%	**1.91**	0.134
Disease-drug	42.12%	**4.36**	0.222

As shown in Table 4, among extracted D1 →D2 pairs with mapped disease names between D1 →D2 pairs and disease-gene pairs, 3.79% pairs shared genes as determined by disease-gene pairs from OMIM. The average number of shared genes was 5.36, a significantly higher number than the average of 0.016 for D1-D2 combination pairs (178,503 pairs for the same 598 mapped diseases). We observed a similar trend when the disease-gene associations from the GWAS studies were used. Among D1 →D2 pairs with mapped diseases, 13.64% shared genes, a much higher percentage than the 3.79% resulting when the disease-gene association data from OMIM was used. The average number of shared genes was 1.91, a significantly higher number than the number of 0.134 for D1-D2 combinations.

We also investigated whether D1 →D2 risk pairs were treated by the same drugs. As shown in Table 3, as many as 42.13% of D1 →D2 pairs shared drug treatments. The percentage was significantly higher than the percentages of D1 →D2 pairs that shared genetic components, indicating that two different diseases with risk-specific semantic relationships were treated with the same drugs even though they did not share any common underlying genetic mechanism. The average number of shared drugs between disease risk pairs was 4.36, significantly higher than the number for all disease-disease combinations: 0.222.

In summary, disease-disease risk pairs tend to share common genetic components and to be treated by the same drugs. Among all 34,448 observed disease-disease risk pairs, only a very small percentage of pairs shared any genes, indicating that we can leverage on the observed strong disease-disease risk-specific relationships to discover underlying novel genetic mechanisms. Similarly, a large percentage (about 58%) of D1 →D2 risk pairs don’t share any drug treatments yet, indicating the usefulness of the extracted D1 →D1 risk pairs in both drug target discovery and drug repositioning.

### Disease-disease pairs with similar risk or effect disease profiles tend to share both genes and drugs

**Figure 3:**
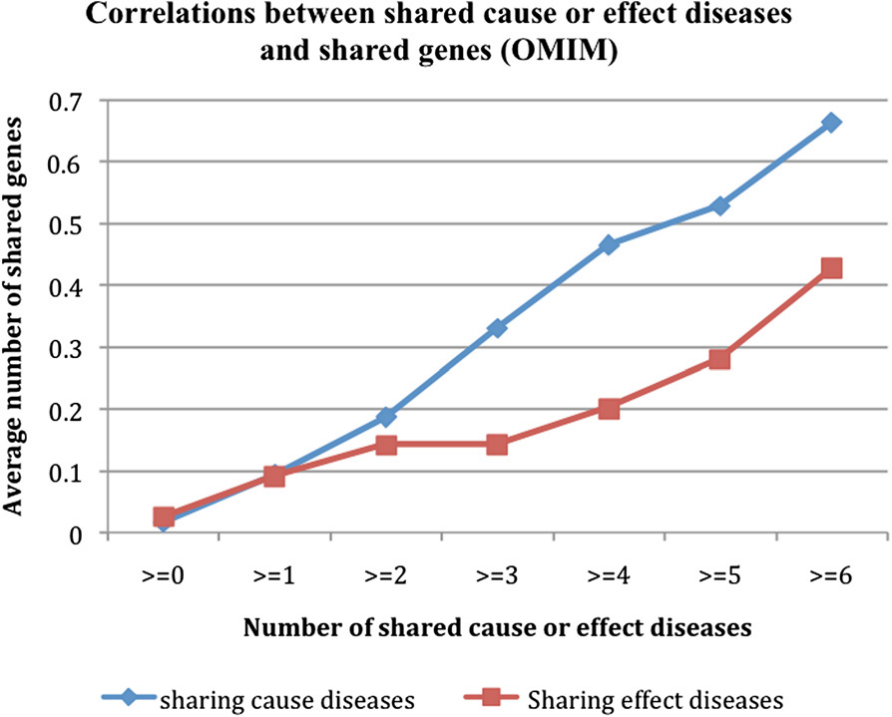
Correlations between disease-disease pairs with shared risk or effect diseases and their associated genes (OMIM).

In the previous section, we showed that a disease and its predisposing diseases (direct D1 ←D2 risk relationship) tended to share both genes and drug treatments. In this section, we investigated whether two diseases with shared risk diseases or effect diseases also shared any genes or drug treatments. As shown in Figure 3, a strong positive correlation between shared risk diseases or effect diseases and shared genes was evidenced. For example, the average number of shared genes for all D1-D2 combination pairs was 0.017 (≥0). The number significantly increased to 0.664 for pairs that shared at least six risk diseases and to 0.428 for pairs that shared at least six effect diseases (≥6). In addition, the correlation was stronger for disease-disease pairs with shared risk diseases than pairs with shared effect diseases.

**Figure 4:**
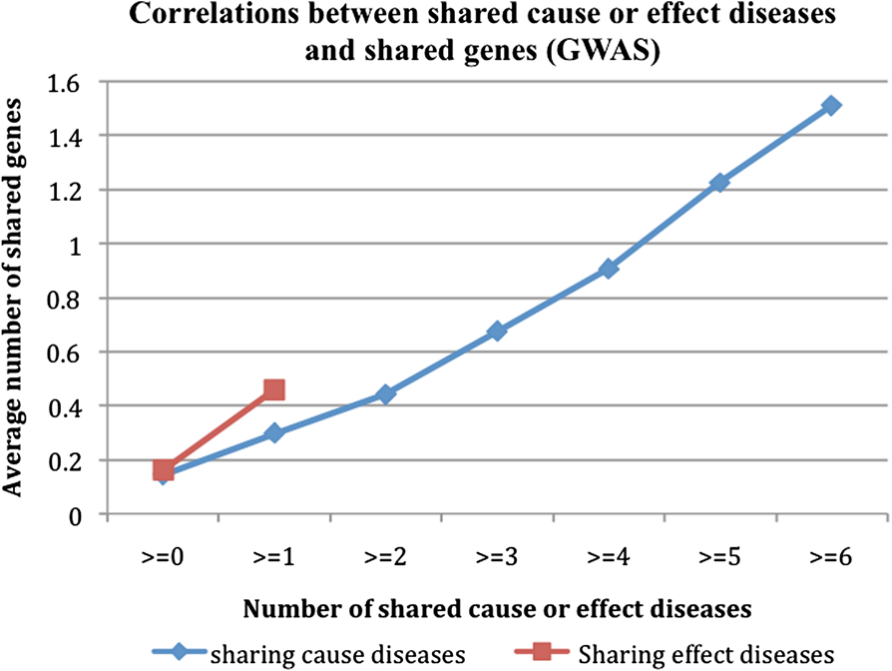
Correlations between disease-disease pairs with shared risk or effect diseases and their associated genes (GWAS).

We observed a similar positive correlation between disease-disease pairs with shared risk diseases or effect diseases and shared genes when the disease-gene associations from the GWAS studies were used (Figure 4). The average number of shared genes for all D1-D2 combination pairs was 0.143 (≥0). The number significantly increased to 1.511 for pairs that shared at least six risk diseases (≥6) and to 0.459 for pairs that shared at least one effect disease (≥1). However, not many D1-D1 pairs (with disease names mapped to disease-gene pairs in GWAS) shared more than one effect disease.

**Figure 5:**
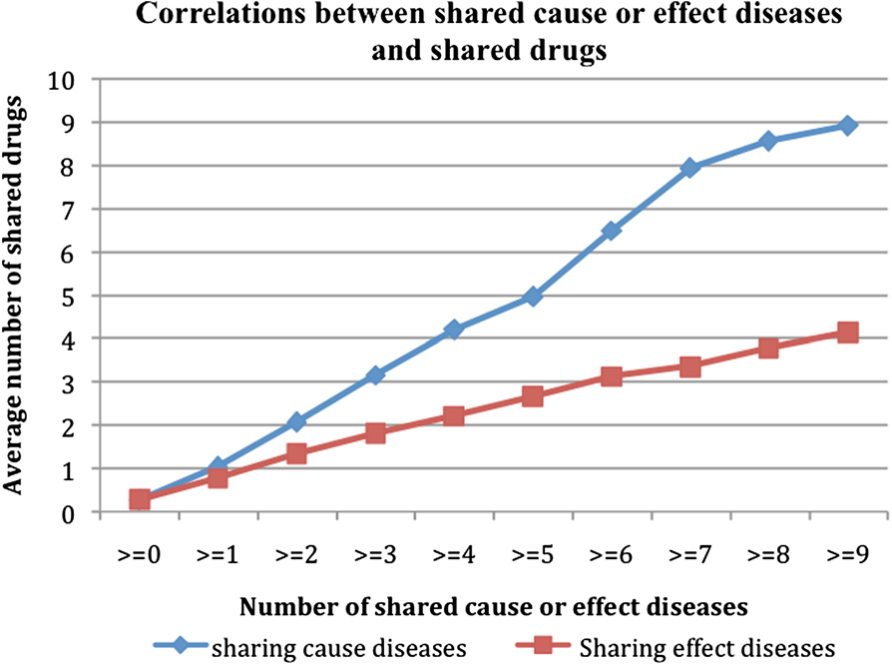
Correlations between disease-disease pairs with shared risk or effect diseases and their associated drugs.

A strong positive correlation between D1-D2 pairs with shared cause or effect diseases and shared drugs was evidenced (Figure 5). The average number of shared drugs for all D1-D2 pairs was 0.273. The number significantly increased to 8.923 for pairs that shared at least nine risk diseases (≥9)and to 4.145 for pairs that shared at least nine effect diseases (≥9). Similar to the correlations when disease-gene associations from OMIM were used, the correlation was stronger for D1-D2 pairs with shared risk diseases than for pairs with shared effect diseases.

### Risk graphs for obesity and type 2 diabetes

**Figure 6:**
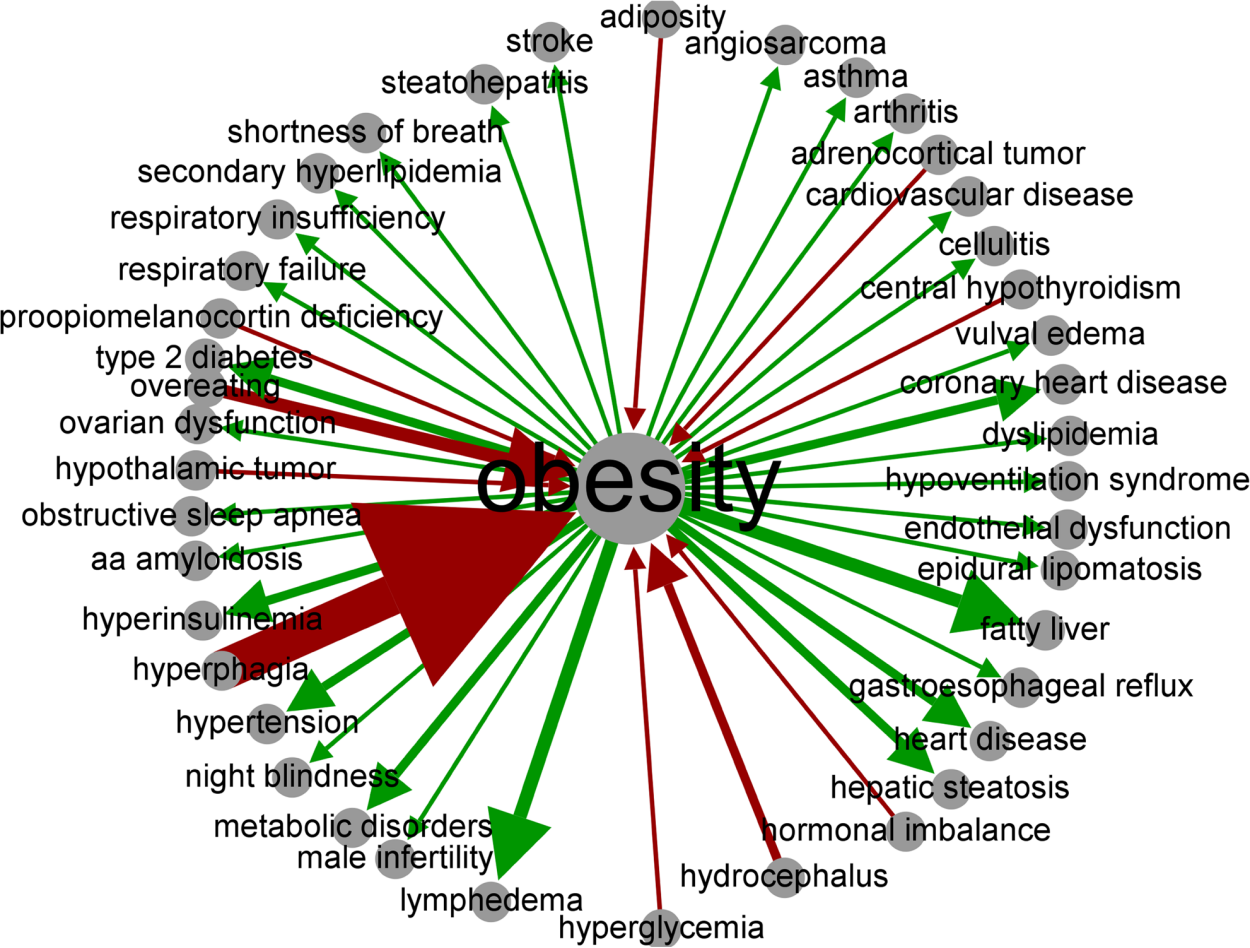
Weighted risk graph directly related to obesity.

**Figure 7:**
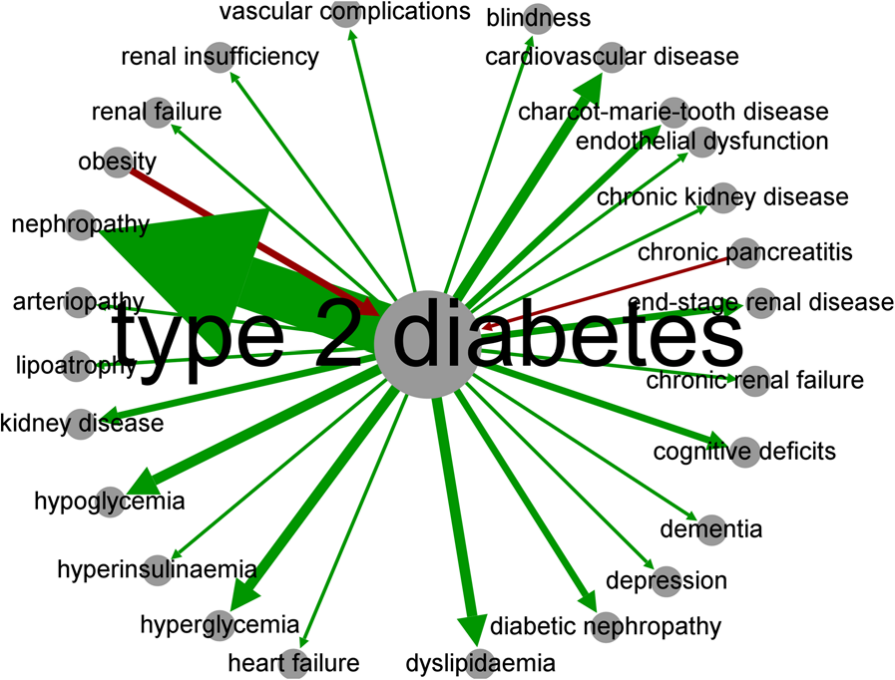
Weighted risk graph directly related to type 2 diabetes (T2D).

In order to visualize the disease risk relationship knowledge represented in dRiskKB, we plotted one weighted risk graph for obesity (Figure 6) and one for Type 2 Diabetes (T2D) (Figure 7). The edge weight was determined by the ranking scores of pairs (“Pair Ranking”). A total of 55 diseases caused or were caused by obesity. The top predisposing disease identified for obesity (D1 →obesity) was *hyperphagia*, a serious eating disorder defined as an extreme, insatisfied drive to consume food. The second-most predisposing disorder for obesity was identified as *overeating*. On the other hand, the top five diseases caused by obesity (obesity →D2) were determined to be *fatty liver*, *metabolic disorders*, *coronary heart disease*, *hypertension*, and *respiratory failure*.

A total of 35 diseases were directly linked to T2D in the dRiskKB (Figure 7). The top one predisposing disease for T2D (D1 →T2D) was identified as *obesity*. Another predisposing disease for T2D was determined to be *chronic pancreatitis*. The top five diseases caused by T2D (T2D →D2) were *nephropathy*, *hypoglycemia*, *cardiovascular disease*, *cognitive deficits*, and *Charcot-Marie-Tooth disease*.

Some diseases have more complicated risk graphs than that of obesity or T2D. For example, hypertension is a condition with a total of 809 directly linked diseases that either cause or are caused by hypertension (graphs not shown).

## Discussion

We built dRiskKB, a disease-disease risk relationship knowledge base by developing an iterative, semi-supervised learning approach to extract a large number of disease-disease risk pairs from the over 21 million published MEDLINE records currently available. Diseases in dRiskKB were linked to their known genes, SNPs, and drug treatments. We also systematically analyzed the relationships between disease-disease risk pairs and disease-associated genes as well as drug treatments. To the best of our knowledge, dRiskKB is the first large-scale disease risk relationship knowledge base built from the large corpus of published biomedical literature.

Nevertheless, our study has several limitations and can be greatly improved in future studies. First, the 26 patterns were selected from top-ranked patterns by manually removing non-disease-risk-specific patterns such as “D1 and D2” and “D1 or D2.” The limitation is that even though it is obvious that these patterns are non-disease-risk-specific, it is difficult to test it formally. Second, even though dRiskKB is precise and consists of a total of 34,448 disease-disease risk pairs, one of the major limitations is that we could not assess its coverage due the lack of a gold standard. Third, currently, dRiskKB only contains the risk relationships among diseases. Many factors other than disease can contribute to the risk of a disease, including genes (e.g. APOE, BRCA1), chemicals (e.g. exposure to benzene, estrogen, aflatoxins), life styles and behavior (e.g. smoking, alcohol use, physical inactivity, excess salt intake), family history, ethnicity, age, gender, and even microbiome in the human body. Automatic extraction of the risk-specific relationship between diseases and these risk factors is a highly challenging task since no specific lexicon of these risk factors currently exists, yet such an entity is required by most information extraction systems for relationship extraction. Fourth, the risk relationships among diseases are often context-sensitive. For example, in the sentence “**Depression ***is a risk factor for ***coronary artery disease** in **men**,” the disease-disease-population triple “*depression CAUSE coronary artery disease IN men*,” rather than the disease-disease pair “*depression-coronary artery disease*,” better captures the context (patient)-specific risk relationships among diseases. Another example is “**hypertriglyceridaemia ***is a risk factor for ***coronary artery disease** in **diabetic populations**,” where triple ‘hypertriglyceridaemia-coronary artery disease-diabetic populations’ better captured the risk relationship between the two diseases. In one of our previous studies, we developed a combined machine learning and NLP approach to accurately extract clinical trial participant information, including demographics, trial size, disease, or symptoms and their descriptors from RCT abstracts [44]. In our future study, we will improve dRiskDB by automatically extracting patient characteristics from sentences.

The majority of extracted D1 →D2 risk pairs (96.21% based on OMIM and 86.36% based on GWAS) don’t share any known genes. At least three factors may account for this. First of all, not all disease-associated genes have been discovered so far. The disease-gene associations from OMIM or GWAS may only cover a small percentage of all disease-associated genes. Second, we only compared the direct gene overlap. It is possible that common genetic pathway or function modules (not necessarily the same genes) are responsible for the observed D1 →D2 risk semantic relationships. Third, non-genetic factors such as environmental factors, diet or socioeconomic status may be responsible for the observed risk relationships among diseases. The facts that D1 →D2 risk pairs shared significantly more genes than all disease combinations and that only a small percentage of the large number of observed D1 →D2 pairs shared any genes provided both motivation and opportunity for developing phenotype-driven network-based approaches to leverage on the observed strong risk-specific semantic relationships among diseases in discovering novel disease candidate genes.

## Conclusions

In this study, we present a semi-supervised approach in order to build a large-scale and precise disease-disease risk relationship knowledge base (dRiskKB). The newly created dRiskKB consisted of a total of 34,448 unique D1 →D2 risk pairs representing 12,981 diseases with each linked to its associated genes and drugs. We have shown that diseases and their risk diseases as well as diseases with similar risk profiles tend to share both genes and drugs. This unique dRiskKB, when combined with existing phenotypic, genetic, and proteomic datasets, can have profound implications in our deeper understanding of disease etiology and in rapid drug repositioning.

## Data availability

The extracted disease-disease risk pairs as well as their associated genes and drugs, the 26 patterns that were used in constructing dRiskKB, and the dataset of curated sentences for pattern precision evaluation are available at: http://nlp.case.edu/public/data/dRiskKB. The curated disease lexicon was created by ThinTek and can be obtained by contacting QuanQiu Wang at qwang@ThinTek.com. We plan to update dRiskKB semi-annually.

## Competing interests

The authors declare that they have no competing interests.

## Authors’ contributions

Xu and Wang have jointly conceived the idea, designed and implemented the algorithms. All the authors have participated in study discussion and manuscript preparation. All authors read and approved the final manuscript.
